# Deregulation of Transcriptional Enhancers in Cancer

**DOI:** 10.3390/cancers13143532

**Published:** 2021-07-14

**Authors:** Fatemeh Mirzadeh Azad, Yaser Atlasi

**Affiliations:** 1 Department of Life Sciences and Systems Biology, University of Turin, 10123 Turin, Italy; Fatemeh.mirzadehazad@unito.it; 2 Patrick G. Johnston Centre for Cancer Research, Queen’s University Belfast, Belfast BT9 7AE, UK

**Keywords:** enhancer, cancer, epigenetic, stem cell, plasticity

## Abstract

**Simple Summary:**

One of the major challenges in cancer treatments is the dynamic adaptation of tumor cells to cancer therapies. In this regard, tumor cells can modify their response to environmental cues without altering their DNA sequence. This cell plasticity enables cells to undergo morphological and functional changes, for example, during the process of tumour metastasis or when acquiring resistance to cancer therapies. Central to cell plasticity, are the dynamic changes in gene expression that are controlled by a set of molecular switches called enhancers. Enhancers are DNA elements that determine when, where and to what extent genes should be switched on and off. Thus, defects in enhancer function can disrupt the gene expression program and can lead to tumour formation. Here, we review how enhancers control the activity of cancer-associated genes and how defects in these regulatory elements contribute to cell plasticity in cancer. Understanding enhancer (de)regulation can provide new strategies for modulating cell plasticity in tumour cells and can open new research avenues for cancer therapy.

**Abstract:**

Epigenetic regulations can shape a cell’s identity by reversible modifications of the chromatin that ultimately control gene expression in response to internal and external cues. In this review, we first discuss the concept of cell plasticity in cancer, a process that is directly controlled by epigenetic mechanisms, with a particular focus on transcriptional enhancers as the cornerstone of epigenetic regulation. In the second part, we discuss mechanisms of enhancer deregulation in adult stem cells and epithelial-to-mesenchymal transition (EMT), as two paradigms of cell plasticity that are dependent on epigenetic regulation and serve as major sources of tumour heterogeneity. Finally, we review how genetic variations at enhancers and their epigenetic modifiers contribute to tumourigenesis, and we highlight examples of cancer drugs that target epigenetic modifications at enhancers.

## 1. The Epigenetic Foundation of Tumour Plasticity

### 1.1. Tumour Plasticity and Heterogeneity

Intratumour heterogeneity is one of the main features in cancer. It entails the presence of phenotypically and functionally distinct subpopulations of cells that can affect processes such as tumour invasion, metastasis or therapy resistance [1]. The impact of tumour heterogeneity is more evident when genetic markers are used as indicators of therapy regimens. For example, in oestrogen/progesterone-positive breast tumours, hormonal castration causes a primary shrinkage of the tumour mass; however, in many cases, recurrence is seen due to the presence of hormone refractory cells in the primary tumour [2,3,4,5]. The classic view of tumour heterogeneity is based on the “clonal evolution” theory of cancer, which suggests that intratumour heterogeneity is caused by the accumulation of genetic mutations in either the tumour bulk or its surrounding stroma, followed by the selection of clones that gain survival advantages. However, intratumour heterogeneity may also arise from processes that induce cell-state transitions without changing the genetic landscape. Such cell plasticity is directly controlled by epigenetic mechanisms and can provide opportunities to reverse the tumour cell behaviour, for example, via differentiation-inducing therapies.

One of the processes that underlies tumour heterogeneity is the cellular differentiation of tumour cells that harbour a stem cell capacity. These cancer stem cells (CSCs) generate committed cells with a spectrum of phenotypes, that all share the same genetic background [6,7], making CSCs one of the main culprits of tumour heterogeneity [8,9]. Recent studies suggest that stemness can be considered as a “biological state” in which cells can enter or exit, indicating a robust cell plasticity within tumours [10,11]. Here, tumour cells can gain stem-like or differentiated phenotype depending on the intrinsic genetic triggers or the external environmental cues. For example, in luminal breast tumours, in which the majority of cells have epidermal characteristics, a small fraction of cells expresses the mesenchymal/basal markers (e.g., CD44) and show resemblance with the normal mammary stem cells. Of note, a homogenous population of luminal tumour cells, sorted based on low levels of CD44(CD44^low^), can regenerate the tumour bulk that contains CD44^high^ cells (constituting 10% of the tumour bulk). Thus, the luminal breast tumour cells can undergo de-differentiation to obtain a more basal CD44^high^ phenotype, suggesting a strong plasticity of cells within the tumour [11].

EMT is another major mechanism fuelling tumour plasticity [12,13,14]. EMT is a process of cell-state transition in which cells lose their apicobasal polarity and gain a mesenchymal-like phenotype. In cancer, this transition includes a spectrum of states and can result in divergent clusters of hybrid cells showing a mixture of traits from the two ends of the epithelial–mesenchymal spectrum [15,16]. Recent findings indicate that this ‘hybrid state’ has a transcriptional profile with similarities to cancer stem cells and is governed by activation of the EMT-inducing factors similar to SNAIL and the stemness maintenance pathways, such as the canonical WNT signalling. A common feature of CSCs and cells in the hybrid state is their high tumourigenicity and stemness potentials, making EMT a major contributor to cancer [17,18,19]. Interestingly, forcing cells into acquiring a fully differentiated epithelial or mesenchymal phenotype leads to a drastic drop in tumourigenicity. For example, the constitutive overexpression of the mesenchymal master regulator ZEB1 in breast cancer cells with a hybrid-state phenotype induces a full mesenchymal profile and a decrease in tumourigenicity that is accompanied by a switch to non-canonical WNT signalling [17].

Another main source of tumour heterogeneity is the spatial organisation of tumour cells and their interactions with the tumour stroma. The sub-cellular distribution of ß-catenin is a classic example of tumour heterogeneity affected by the environmental cues. The nuclear enrichment of ß-catenin (a known sign of active WNT signalling) is particularly visible at the invasive front of *APC*-mutant colorectal tumours, whereas the proliferating cells of the tumour bulk show a more membranous ß-catenin localisation [20]. This observation is partly due to the gradient of growth factors and the cytokines secreted by the tumour microenvironment. The uneven diffusion of these signalling proteins (such as HGF and WNT ligands) can divide the tumour into different foci. Here, cells with a mesenchymal characteristic that show nuclear distribution of ß-catenin are located at the tumour periphery and are in close contact with the tumour stroma [21,22].

In addition to phenotypic variation, our understanding of the extent of tumour heterogeneity has tremendously increased by using technologies that reveal features of tumour cells at the single cell level. One such approach is based on investigating the gene expression profiles by high-resolution single cell RNA-seq (scRNA-seq) analysis [23]. For example, a recent study of a BRCA1-null model of breast cancer indicates that tumour cells with a similar genetic background can cluster into different subpopulations that have distinct gene expression profiles. In this regard, the upregulation of cell cycle regulators (e.g., *BIRC5*, *TYMS* and *MKI67*) is only observed in a cluster of highly proliferative cells and not in the cell cluster that exhibits a progenitor-like phenotype (with the expression of the basal cell markers such as *KRT14*, *IGFBP5*, *WNT10A*). These observations suggest that cancer therapies that target cell proliferation may be less effective in eradicating the quiescent progenitor-like populations in these tumours [24,25,26].

### 1.2. Enhancers, the Epigenetic Playground

**Figure 1:**
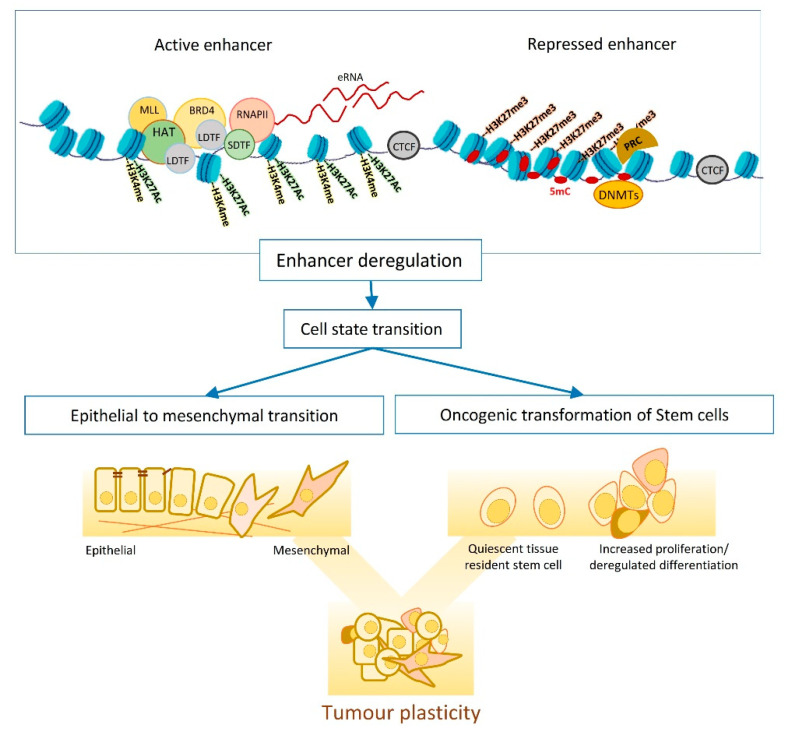
Schematic representation of the general features of active and inactive enhancers. The intra-tumour heterogeneity may arise from differentiation of cancer stem cells or EMT, two main processes that involve cell state transition and epigenetic reprogramming of transcriptional enhancers.

Given the strong cell plasticity observed within tumours, a question that emerges is how these reversible cell-state transitions are controlled at the epigenetic level. In this section, we discuss transcriptional enhancers that are among the main sites of epigenetic regulation. Enhancers are *cis* regulatory elements (CREs) that function as information routers connecting the upstream signalling pathways to the downstream genes [27]. It is suggested that enhancers regulate their target gene expression regardless of distance and orientation. Enhancers interact with transcription factors (TFs), that induce chromatin accessibility at these sites, and are often decorated with various histone marks (Table 1), that are assessed by chromatin immunoprecipitation (ChIP)-based assays. For instance, H3K4me1 (mono methylation of lysine 4 at histone 3) is a general marker of poised and active enhancers, H3K27ac (acetylation of lysine 27 at histone 3) is mainly associated with active enhancers, while H3K4me3 (tri methylation of lysine 4 at histone 3) is enriched at active promoters and H3K27me3 (tri methylation of lysine 27 at histone 3) denotes poised or repressed enhancers (Figure 1) [28,29,30,31]. Some enhancers are also transcribed and give rise to non-coding RNAs known as enhancer-RNA (eRNA) that can be used to assess enhancer activity [32,33,34]. Therefore, the profile of active enhancers ensures the spatiotemporal expression of target genes to sustain cell identity. The pattern of the distribution, combination and sequence degeneration of TF binding sites (TFBSs) control the regulatory output of enhancers [35]; thus, the deregulation of signalling effector TFs, transcription co-factors, and disruption of DNA sequence can affect enhancer function downstream of cell signalling (Figure 1).

Using the above-mentioned features, an experimental approach based on a combination of ChIP-seq for histone modifications and TF-binding, RNA-seq for tracking the transcriptional activity, and DNaseI-seq or ATAC-seq approaches for mapping the chromatin accessibility, can be used to identify functional enhancers. Furthermore, by scrutinising the DNA sequence at enhancer sites, for example, using motif search at open chromatin regions decorated by H3K27ac and H3K4me1, the cell-state specific TFs can be annotated and further validated by immunoprecipitation approaches. For instance, applying motif discovery at RAS-dependent open chromatin regions shows the enrichment of AP-1 and Stat92E at enhancers that gain activity downstream of RAS signalling. In this case, the oncogenic recruitment of Stat92E to regulatory elements deems necessary for tumourigenesis and can be further validated by ChIP and mutagenesis experiments [54,55,56,57].

The exact sequence of events that leads to enhancer activation is not well established; however, pioneering factors and lineage defining TFs (LDTFs) can access the condensed chromatin regions and can act as the initial step for enhancer activation. These TFs recruit the epigenetic modifiers, e.g., the histone acetyl transferases CBP/P300 and/or the mono methyl transferases MLL3/MLL4, to deposit H3K27ac and H3K4me1 at enhancers. These active enhancer marks are then recognised by epigenetic readers such as BRD4 that recruit transcription coactivator, the Mediator complex and the RPOL2 transcription machinery to maintain the expression of target genes [58,59].

In order to activate transcription, enhancers need to interact with their target promoters. These interactions are mediated by proteins such as Cohesin that enable chromatin looping. Data from high resolution chromatin mapping shows that the network of enhancer–promoter (EP) interaction changes in tumour cells and contributes to cancer progression. These alterations can be caused by the deregulation of proteins involved in chromatin organisation and looping (e.g., BRCA1 in luminal breast cells [60] or Cohesin in leukaemia [61]), epigenetic modifiers that control enhancer activity (e.g., EZH2 [62]), or structural genetic variants that bypass enhancer-flanking insulators, leading to enhancer hijacking [63]. Oncogenic TFs can also contribute to establishing specific EP interactions in tumours. For example, in prostate cancer, cancer-specific EP interactions involve enhancers that are enriched for and are activated by oncogenic TFs such as FOXA2. These FOXA2-dependent enhancers engage in EP interactions that are specific to tumour cells and influence the expression of key oncogenes, such as the Androgen Receptor (AR) and DLX1 in prostate cancer [64]. Chromatin looping and EP interactions are also among the targets of cancer therapy. For example, in endocrine-resistant oestrogen receptor (ER)-positive breast tumours, inducing global DNA hypomethylation can overcome therapy resistance. This treatment demethylates and activates the ER-responsive enhancers that in turn establish new interactions with the promoters of tumour suppressor genes, leading to their activation and the suppression of tumour growth [65].

New advances in single cell technologies reveal the heterogeneity of enhancer activity within tumours. For example, mapping chromatin accessibility by scATAC-seq across glioblastoma cells not only confirmed the presence of stem-like cells within the tumours, but also revealed further diversity within the CSCs population. Here, all CSCs share a fraction of active regions corresponding to genes involved in self-renewal and tumourigenicity; however, a fraction of accessible chromatin sites also showed diversity between CSCs. These sites include motifs for factors that affect invasion (FOXD1 and ALDH1A3), response to immune signalling (SP1) and neural commitment (OLIG2, AHR). Combined with scRNA-seq, these findings confirm the heterogeneity even within the tumour-initiating CSCs and highlight the potential challenges in targeted therapies [66,67].

The activity of enhancers can be also regulated by DNA methylation that is deposited at cytosine residues and, based on the genomic location and the co-occurrence of other epigenetic marks, can have a positive or negative impact on transcription. Generally, CpGs methylation at promoters and enhancers is followed by inactivating histone methylation marks and the formation of a condensed chromatin state [68], whereas gene-body methylation shows a positive correlation with transcription [69]. The global hypomethylation, e.g., via mutation in DNMTs, can potentiate the ectopic expression of oncogenes [52]. For example, studies in lymphoma indicate that the hypomethylated fraction of a genome usually harbours genes and CREs that are related to proliferation, differentiation, and negative regulators of P53 pathway [70]. However, an elevated level of DNMT can also contribute to tumourigenesis through the inactivation of tumour suppressor genes [53]. In breast cancer, for instance, the upregulation of DNMTs is necessary for tumour progression; here, the cancer stem cell subpopulation relies on DNMT1 to hypermethylate and suppress *ISL1* that functions as a negative regulator of self-renewal in mammary stem cells and plays a tumour suppressor role in breast cancer [71,72].

Recent studies indicate that DNA methylation of cis-regulatory elements is highly heterogeneous within the tumour bulk. Spatial sampling of breast cancer cell populations revealed a divergent profile of DNA methylation across the tumour that is mainly detected at genes such as *GSTP1*, *FOXC1*, *ABCB1*, *PTEN*, and *TGM2* that contribute to drug resistance. This heterogeneity of DNA methylation is further increased after cancer therapy, in which a specific cluster of stem-like cells repopulates the tumour mass. Heterogeneity in DNA methylation is also observed in genes that regulate the stem cell quiescence (e.g., *SOX9*, *ALDH1L1*, *WNT5A* and *HOPX*), and that are hypomethylated in a small fraction of CSCs [73]. Applying multi-region sampling in prostate tumours also detected a differential pattern of methylation at distal regulatory elements of tumour suppressor genes such as *PTEN*, *TP53*, and *GSTP1*. Moreover, a heterogeneous DNA methylation was also observed at AR-responsive enhancers across the tumour bulk, generating a cluster of cells with different sensitivity to androgen exposure. This heterogeneity could fuel the later clonal evolution and hormone resistance in tumours [74].

## 2. Enhancer Dynamics and Tumour Plasticity:

### 2.1. Enhancer Dynamics and Adult Stem Cell Differentiation

A common route to tumourigenesis is epigenetic deregulation, particularly in adult stem and progenitor cells that are the cornerstone of tissue homeostasis (Figure 1). Haematopoiesis is one of the main paradigms for studying stemness in normal homeostasis and cancer given the well-established cellular hierarchy in the haematopoietic system [75,76,77]. During haematopoiesis, HSCs are found in a relatively quiescent state, whereas the blood cell re-population is mainly driven by proliferative progenitors. In cancer, however, either the normal resident HSC gain proliferative features, or the progenitor cells go through a de-differentiation process. In line with this, the re-activation of an epigenetic repertoire of progenitors and stem cells has been observed in cancer. For instance, HMGN1, a DNA binding protein that regulates chromatin accessibility, serves as one of the main modulators of chromatin architecture in HSC and myeloid progenitors, and is commonly amplified in myeloid malignancies. In myeloid progenitors, HMGN1 is crucial for cell state maintenance, as it regulates the chromatin accessibility and H3K27ac deposition by P300/CBP at HOX loci. Overexpressing HMGN1 in progenitor cells impairs their differentiation, promotes their proliferation, and causes a global increase in chromatin accessibility. The overexpression of HMGN1 therefore leads to the upregulation of oncogenes and loss of lineage-specifying regulators, such as C/EBPα, resulting in an expression profile similar to that of leukaemia stem cells [78,79,80]. The contribution of deregulated enhancers to neoplastic transformation is more evident in aging HSCs that accumulate mutations in epigenetic regulators. During aging, enhancers that control the differentiation, homeostasis, and apoptosis of myeloid/erythroid cells lose H3K4me1 and H3K27ac marking. This loss of active epigenetic marks represses the expression of tumour suppressor genes, such as *KLF6*, *BCL6*, and *RUNX3*, leading to an increased susceptibility to cancer. Not surprisingly, the same repression pattern is also observed in cancer stem cells. Moreover, regulatory elements associated with potential oncogenes, such as *GATA2*, *GFI1B*, and EGR1, also gain active histone marks in aging HSCs [47]. Thus, the aging-related changes in the enhancer landscape appears to mirror some of the oncogenic alterations observed in CSCs, hinting to the notion that aging stem cells might have an increased chance of neoplastic transformation.

Switching between enhancers in different developmental states is another strategy that cells use to tailor their gene expression profiles. In haematopoietic stem and progenitor cells, alternative elements that engage in enhancer switching have different compositions of TF binding sites that include various differentiation regulators (such as MYB, FLI1, LMO2, and RUNX1) and signalling-dependent TFs. This unique array of TF-binding sites and the differential expression of TFs determines the state-specificity and the level of enhancer activity. Interestingly, the deregulation of these TFs is often detected in transformed cells, leading to enhancer switching and the usage of elements with more oncogenic impacts. In line with this, the expression of GATA2 and MYC in haematopoietic malignancies is controlled by the re-establishment of a series of enhancers that normally function in HSCs, further highlighting the contribution of stem-state-specific enhancers to cancer [81,82,83].

The intestine is another tissue with rapid turnover and dynamic cell plasticity. Transcriptional analysis of the stem-like sub-population of colorectal tumours indicate their similarity with LGR5+ stem cells residing in the intestinal crypt. Thus, understanding the epigenetic mechanism controlling the plasticity in normal stem cell can shed light on the contribution of stem cells to tumourigenesis and intratumour heterogeneity [84,85]. Under normal homeostasis, the highly proliferative LGR5+ stem cells support the continuous turnover of the intestine epithelium. Upon injury and when the stem cell population is depleted, other reserve stem cells (such as Bmi1^+^, Hopx^+^, or Lrig1^+^ cells) that normally have low proliferation rate can replenish the LGR5+ stem cells. In addition, more committed secretory and enterocyte (absorptive) progenitors can also de-differentiate to LGR5+ stem cells to support intestine regeneration upon injury [85,86,87,88]. The profound cell plasticity in the intestine suggests the dynamic reprogramming of enhancers during stem cell (de)differentiation. Using the H3K3me2 that marks active and poised enhancers, a striking similarity was observed between LGR5+ stem cells, secretory progenitor, and absorptive progenitor cells. These findings indicate that many enhancers that are active in progenitor cells are already primed in LGR5+ stem cells. A large number of these enhancers also show the H3K27ac active mark in stem cells and progenitor cells, indicating a broadly permissive chromatin among intestinal crypt progenitors [89]. In the tumour context, the dynamic of histones modifications is vital to the CSC pool and is usually controlled downstream of stemness regulatory pathways. For example, it is known that LGR5^+^ intestinal cancer stem cells are dependent on canonical WNT signalling. In these cells, nuclear ß-catenin needs to cooperate with MLL1 methyl transferase for the activation of WNT-responsive elements. MLL1 antagonizes the deposition of repressive H3K27me3 by PRC2 and marks the ß-catenin-bound regions with activator H3K4me3 that controls genes such as *LGR5*, *SMOC2* and *IGFBP4*, which are needed for maintaining stemness identity [45].

The analysis of DNA methylation in intestinal stem cells and their differentiated progenies also demonstrates that only a few promoters and enhancers change their DNA methylation status during stem cell differentiation. Thus, in contrast to ESCs and haematopoietic stem cells, intestinal stem cell differentiation does not require DNA methylation for the stable lock of gene expression. In addition, many differentiation-associated genes already show hypomethylated status in intestine stem cells, indicating an epigenetic priming of stem cells [90]. In contrast to normal homeostasis, alteration in DNA methylation is one of the primitive changes in neoplastic LGR5+ intestinal stem cells downstream of oncogenic *APC* mutation. Although the pattern of DNA methylation does not change drastically during normal differentiation, APC^ko^ stem cells acquire a distinct methylation landscape. The impaired methylome is mainly observed in intergenic and intronic regions, affects genes that control stem cell self-renewal and attenuates stem cell differentiation. This impeded commitment to differentiation results in the accumulation of LGR5+ intestinal stem cells in tissues after the loss of *APC* and hyperactivity of WNT signalling. In fact, suppressing de novo methyl transferases in APC negative intestinal organoids could sensitize the LGR5+ stem to differentiation stimuli. These data confirm the importance of DNMTs activity in transformed stem cells and its contribution to tumourigenesis. Aside from hindering stem cell differentiation, deregulated DNA methylation can also lead to the reactivation of transposable elements (TE). Evoked due to hypomethylation, the transposition of TEs predisposes the cells to genomic instability, which contributes to cancer progression by increasing the level of chromosomal aberrations and genomic gain at oncogenes [91,92].

### 2.2. Enhancer Dynamics and EMT

EMT is one of the main mechanisms fuelling the tumour plasticity and heterogeneity and entails dynamic changes in transcriptional and epigenetic landscapes (Figure 1) [12,93]. Studying EMT in normal and transformed cells indicates dependency on global epigenetic reprograming, that starts with extracellular cytokine or the induction of EMT master regulators (e.g., *TWIST* and *SNAIL*). DNA methylation is one of the waves of epigenetic changes that is pivotal for the EMT cell state transition, as suppressing DNMTs (e.g., by chemical inhibition of their functions) impairs EMT, even when the EMT-inducing signal persists [93,94,95]. The reprogramming of DNA methylome starts shortly after EMT induction (e.g., by TGFB) and lasts until the cells commit to a mesenchymal state. In transitioning cells, hyper methylation occurs at CpGs containing regulatory regions that associate with epithelial identity and cell cycle progression, resulting in chromatin condensation at these sites. Histone modifications are another layer of epigenetic regulations that dynamically change in response to an elevated level of EMT master regulators such as SNAIL [48,96,97]. SNAIL mainly functions as a transcription repressor and modulates the loss of H3K27Ac and H3K4Me3, as well as the enrichment of H3K27me3 by recruiting corresponding chromatin modifiers to the promoter of epithelial markers. However, later on in the transition, SNAIL induces positive histone marks (H3K4Me3 and H3K4Me1) on the promoter of mesenchymal genes to support the mesenchymal commitment [48].

Among different extracellular signals, TGFB is one of the most potent inducers of EMT that triggers a global epigenetic reprogramming in target cells. Several signalling cascades function downstream of TGFB to transmit the signal to target genes. For example, in mammary epithelial cells, ERK signalling plays a crucial role downstream of TGFB to regulate H3K27ac deposition at enhancers. In this regard, enhancers activated by TGFB are highly enriched for TFs such as GABPA, JUN, RUNX1, and ATF3 that function downstream of ERK signalling and have prominent roles in EMT. Furthermore, when ERK-signalling is inhibited, cells treated with TGFB fail to express early EMT regulators such as HMGA2, ITGA2, and TGFBR1 due to a lack of H3K27ac deposition at corresponding enhancers. Thus, ERK-signalling is necessary for acquiring H3K27 acetylation at enhancers and the induction of the EMT process [98,99]. The TGFB-induced EMT is not always conveyed through canonical TFs (e.g., SNALI or ZEB1). In alveolar carcinoma cells, an unconventional trio of ETS2, HNF4A, JUNB show overexpression in the E/M hybrid phase of EMT and are necessary for the formation of TGFB-induced super enhancers. These super enhancers control the expression of mesenchymal markers such as FOXP1 and CDH2 in transitioning cells. The synergic effect of ETS2, HNF4A, JUNB is crucial for enhancer activity as their suppression impairs the EMT process through loss of enhancer activity [100].

The flow of information from the extracellular EMT inducers to chromatin is not well understood. However, several master regulators of EMT, such as ZEB1 and SNAIL, are shown to control downstream epigenetic modifiers. For example, ZEB1 increases the H3K4me3 deposition at EMT-related regulatory elements by controlling the histone methyl transferase, SETD1B. This regulatory pathway is also involved in colorectal cancer (CRC), in which increased levels of ZEB1 and SETD1B are correlated with high tumour invasion and poor prognosis [46]. Hijacking epigenetic modifiers by EMT regulators is not restricted to epigenetic activators; a case in point is ZEB1, which can suppress the epithelial markers (e.g., E-cadherin) via interaction with HDAC1 [101], BRG1 chromatin remodeller [102] and DNMT [103]. Thus, the combinatorial effects of altered epigenetic activators and repressors set the transcription stage during the EMT process [104]. The EMT master regulators can also control the binding pattern of epithelial TFs at regulatory elements. In CRC, for instance, SNAIL disrupts the activity of epithelial specific enhancers by the transcriptional repression of *FOXA1*. The FOXA pioneering factors are integral to epithelial homeostasis and their chromatin binding is crucial for commissioning the epithelial specific program. Thus, a reduction in FOXA1 at enhancers of key epithelial genes (such as *CDH1*, *EPHB3* and *CDX2*) can lead to decreased H3K4me1 and H3K27ac at these sites and transcriptional changes that favour the mesenchymal transition [105,106].

In CRC, SNAIL also modulates the transcriptional program of WNT signalling in favour of EMT by changing the balance of available WNT effectors and suppressing some of the EMT inhibitors downstream of WNT signalling. Although WNT signalling is known to act as a positive contributor to EMT, it also regulates the homeostasis of normal intestinal epithelium. In this context, some of WNT-responsive regulatory elements enhance the expression of epithelial genes and can negatively impact EMT. One of these WNT-responsive factors is *EPHB2*, a tumour suppressor gene that controls the distribution and organisation of proliferative epithelial cells in normal crypts. The positive regulation of WNT is applied through β-catenin/TCF7L2 binding at the *EPHB2* enhancer. Upon EMT, SNAIL upregulates another WNT effector, LEF1, which competes with TCF7L2 for binding to β-catenin at *EPHB2* enhancer. The β-catenin/LEF1 complex decommissions the EPHB2 enhancer and abrogates its responsiveness to active WNT signalling during EMT [107,108].

EMT regulation can be cell-type specific, and the downstream epigenetic changes can vary, dependent on the EMT stimuli. For example, EMT induction in renal, alveolar and breast immortalised cells reveals that the changes in histone methylation (at H3K27, H3K4 and H3K9) is cell-type specific. This difference in epigenetic regulation is also observed when different EMT stimuli such as TGFB/TNFa or EGF are used. This context-specific epigenetic regulation is associated with different transcriptional outputs. For instance, TGFB treatment causes the downregulation of E-cadherin in all investigated cell lines, whereas the gain of mesenchymal markers such as Vimentin is detected in some cells. Nevertheless, a fraction of regulatory elements is similarly decorated with histone marks in all conditions, regardless of the source of EMT induction. Interestingly, these universal EMT elements control genes that function in extracellular matrix degradation (e.g., *ADAM* and *MMP9*) rather than EMT-related transcription factors [109,110].

## 3. Cancer-Related Genetic Variations at Enhancers

### 3.1. Mutations Affecting the Enhancer Sequence

About 324 million genetic variants are known in the human genome, with an estimated 5 million sites differing between each individual genome and the reference sequence. These genetic variants mainly comprise single nucleotide variation (SNPs) or structural variants, including duplications, insertions, inversions, and translocations. The large fraction of genetic variants are often low-penetrance risk factors that occur at the non-coding genome, including enhancers, promoters, insulators and ncRNAs. Genome-wide association studies (GWASs) indicate that these risk loci show an overrepresentation at, among others, TF binding sites [111] and cell-type-specific regulatory elements, suggesting that many of these risk loci are likely to affect enhancer function. Furthermore, mutations at enhancers can affect epigenetic marking in cancer. Recent studies based on whole genome sequencing (WGS) analysis indicate that active TF binding sites exhibit higher mutation frequency due to the inhibition of DNA repair at these sites when compared to closed chromatin regions [112].

**Figure 2:**
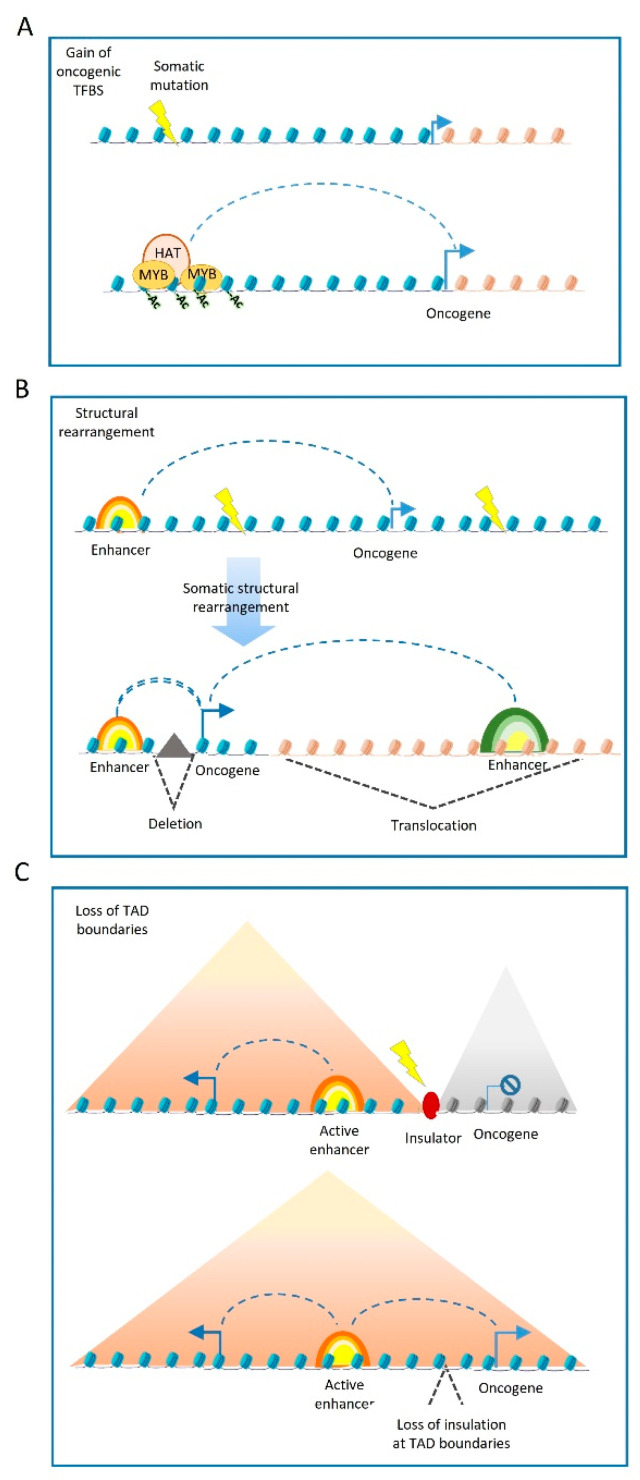
Contribution of mutations that affect enhancer sequence in cancer. (**A**) Somatic mutations can generate novel TF binding sites that recruit epi-modifiers that in turn can deposit epigenetic marks forming putative oncogenic enhancers. (**B**) Structural rearrangement of DNA can increase the expression of oncogenes by assigning ectopic enhancers to oncogenes via chromosomal translocation or by reducing the distance of enhancer to target promoter via deletions. (**C**) Mutations and structural rearrangement can influence the insulator regions and consequently can disturb TAD boundaries that shield oncogenes from nearby enhancers.

In general, genetic variants can be divided into somatic mutations that are not passed to the next generation or germline variants that are inherited from parents and are present in germ cells. Although most somatic genetic variants in cancer occur in the non-coding genome, recent studies from the ICGC/TCGA Pan-Cancer Analysis of Whole Genomes (PCAWG) demonstrate that the number of driver mutations at enhancers is much lower than coding somatic mutations. Identifying driver mutations is usually based on the high recurrence of the variants in cancer or a strong functional impact in tumourigenesis. To identify the impact of non-coding genetic variants, the PCWAG consortium performed WGS in 38 tumour types that included 2658 tumours and matched non-tumour samples. Based on this approach, 13% (785 out of 5913) of all identified driver point mutations are detected at non-coding regions. On average, 4.6 driver mutations per tumour are detected in coding and non-coding DNA, including 2.6 driver point mutations in coding and 1.2 driver mutations in non-coding genomes. Mutations at *TERT* promoter represented the most frequent noncoding driver mutations and comprised ~one third (237 out of 785) of all non-coding DNA mutations. These mutations activate the TERT expression and result in increased telomere length in somatic cells that is associated with tumourigenesis. Among other non-coding mutations that affect tumourigenesis are point mutations at enhancers of *FOXA1* in prostate cancer [27] or mutation at the *ADGRG6* enhancer in bladder cancer [113]. Somatic mutation can also form novel TF binding sites that mark the regions as putative oncogenic enhancers (Figure 2A). This gain of enhancer activity is reported in acute lymphoid leukaemia (ALL), in which a point mutation generated a MYB binding site. This ectopic binding site leads to MYB binding and a consequent increase in H3K27ac, generating an oncogenic super enhancer that induces *TAL1* expression [114]. However, despite the larger fraction of somatic variants occurring in non-coding regions, only a small number of these mutations appear to be driver mutations when compared to the coding-region drivers [115].

In addition to point mutations, somatic structural rearrangements (deletions, duplications, inversions, or translocations) can also affect enhancer function by disrupting enhancer–promoter interactions or by translocating enhancers to the proximity of the target gene, consequently impacting gene expression (Figure 2B). Based on PCWAG data, structural variants can significantly influence gene expression. In general, many structural variants significantly increased gene expression, as was observed for the *TERT*, *MDM2*, *CDK4*, *ERBB2*, *CD274*, *PDCD1LG2*, and *IGF2* loci. However, genetic rearrangements of nearby genes do not always increase the number of interacting enhancers but often lead to a closer proximity of enhancers to target promoters [116].

Somatic structural variants may also disrupt the boundaries of insulated genomic domains and can lead to deregulated gene expression (Figure 2C). Most enhancer–promoter interactions occur within topologically associating domains (TADs) [117] that insulate the regulatory activity of enhancers from neighbouring domains. In this regard, structural variants can lead to TADs fusion, the duplication of TAD-boundaries or complex rearrangements, such as TAD inversions. The PCWAG found that structural variants affecting TAD boundaries occur in 5.0%, 8.5%, and 12.8% of all deletions, inversions, and duplications, respectively. Such examples have been observed in a TAD boundary deletion near the *WNT4* locus in lymphoma, and the *SLC22A2* locus in breast cancer. However, PCWAG results indicate that structural variation at TAD boundaries do not strongly affect the expression of nearby genes; only in 14% of the cases, deletion in a TAD boundary results in the significant expression change of nearby genes [116].

### 3.2. Mutations at Enhancer Regulators

It is important to note that enhancers operate as platforms for the epigenetic machinery. Thus, in addition to genetic variations in cis-regulatory elements, mutations in enhancer-associated proteins can also influence cell state, tumour formation and progression, via altered epi-decoration of enhancers [118,119].

Post-translational modifications at H3K27 are imperative for defining the activity of promoters and enhancers, and variations that influence these modifications can lead to altered transcription. This has been observed for *KDM6* (also known as *UTX1*) and *EZH2,* which are the main demethylase and methyl-transferase of H3K27, respectively; mutations in these factors are frequently detected in cancer and can change the methylation pattern at H3K27 [49,50,120]. For example, in bladder cancer, which has one of the highest frequencies of *KDM6A* mutations, *KDM6* inactivation leads to increased H3K27 methylation at regulatory elements that control the tumour suppressor genes such as *IGFBP3*. *IGFBP3* is a known pro-apoptotic factor and its suppression leads to aberrant cell cycle progression and tumourigenesis [50]. KDM6 also functions as a component of the COMPASS-like complex, which demarcates enhancers by depositing activating H3K4 methylation marks. Thus, in addition to controlling the expression of tumour suppressor genes, the loss of KDM6 can lead to the irregular activity of enhancers that induce the expression of oncogenes such as *KRAS* and *RUNX3* [121].

A precisely regulated pattern of H3K27 acetylation is pivotal for the maintenance of cell homeostasis, as mutations in acetyl transferases are frequently reported in primary [36] and relapsed tumours [37]. Inactivating mutations, ranging from point mutations to deletions, usually affect the HAT catalytic domain and can lead to a decline in histone acetylation at regulatory elements. This decrease in histone acetylation is usually observed at enhancers and promoters that control the transcription of tumour suppressor genes. For instance, the decreased activity of P300/CBP in keratinocytes results in a drastic drop in the expression of a negative regulator of MAPK/ERK signalling, MIG6, and promotes cell proliferation and tumourigenesis [38,42,122]. In some cases, other orthologue proteins can compensate the defected HAT [39]. For example, the CBP-deficient tumour cells depend on P300 to sustain H3K27ac not only at the homeostatic genes but also the oncogenic expression of *MYC* [39]. Of note, the HAT compensation by other orthologous cannot entirely recapitulate the regulatory functions of the defected counterpart, as different HATs engage in distinct protein interactions. Histone acetyl transferases can also acquire point mutations outside the core catalytic domain that can subsequently alter the protein–protein interactions [41]. Falling into this category is the P300 S89A mutation, which disrupts its interaction with β-catenin in intestinal epithelium. Impaired WNT/P300/β-catenin signalling results in the downregulation of genes involved in differentiation, metabolism, and cell–cell interaction. Mice models carrying S89A mutation are highly sensitive to intestinal insults and are predisposed to cancer formation [40].

The other side of balancing the histone acetylation is controlled by histone deacetylases such as HDAC1. A multi-omics study in liposarcoma shows that HDAC1 is mutated in 8.5% of primary tumours. Mechanistically, HDAC1 inactivation leads to the deregulated expression of lineage-defining TFs such as C/EBPα, enhancing the undifferentiated state of tumour cells [123]. In another case, CRC cells that carry a frame-shift mutation at the HDAC2 gene become refractory to anti-proliferative drugs. This loss of HDAC2 correlates with increased histone acetylation at regulatory elements that control various pathways involved in cell proliferation [124,125].

Considering the importance of H3K27 in accepting various activator or inhibitory modifications, mutations that occur at this histone residue can also directly impact epigenetic regulations. Oncogenic missense mutations in H3K27 (e.g., H3K27M) have been reported in several cancers, especially early onset gliomas. This dominant negative mutation that usually affects only one of the H3 coding genes, can affect the expression of onco- and tumour suppressors, and in some tumours (such as low grade glioma) contributes to poor prognosis and overall survival [126]. Mechanistically, di- and tri-methyl deposition on H3 is reduced due to the stalling of PRC2 over H3K27M. Although the mutant histone residue does not impair the recruitment of PRC2, it inhibits the spread of methylation along regulatory elements. Genes that are affected by this methylation impairment are among stemness and lineage-defining regulators that do not require a high level of expression to induce oncogenic transformation [127,128]. In addition, inefficient and aberrant methylation at regulatory elements can interfere with the chromatin landscape and can promote the activation of enhancers. Thus, the presence of this onco-histone results in heterotypic nucleosome formation with reduced methylation and an elevated level of acetylation at the wild-type H3K27. This altered landscape of H3K27 modifications ultimately affects the MAPK and Rho-associated GTPase signalling, that, along with the deregulation of lineage specific genes, contribute to glioma formation [121].

### 3.3. Epigenetic Cancer Drugs that Target Enhancers

**Figure 3:**
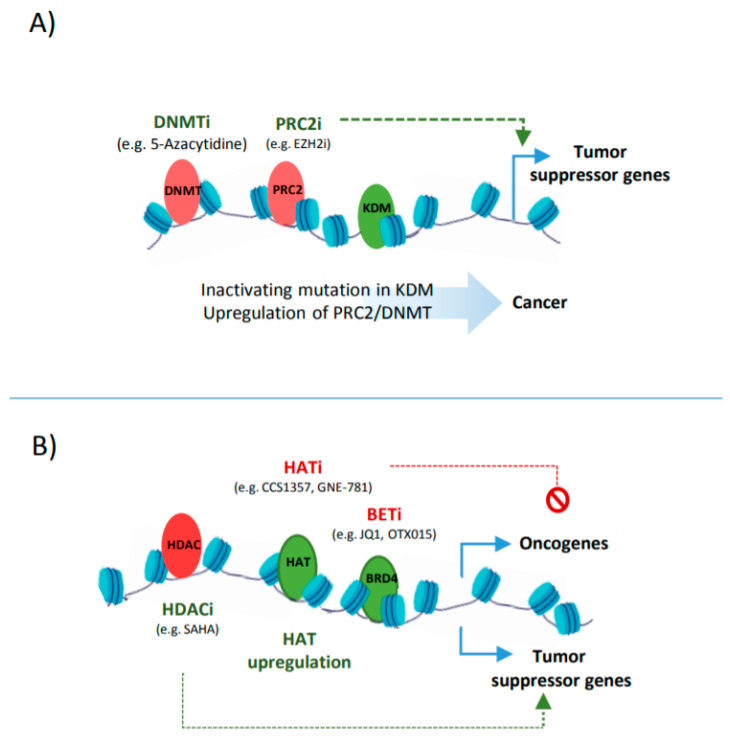
Targeting epigenetic modifiers in cancer by epi-drugs. (**A**) Excessive expression of transcription co-activators such as HATs and BRD4 in tumours leads to upregulation of oncogenes. Inhibition of HATs by CCS1357, GNE-781, JQ1 and OTX015 can abolish their enhancing effect on oncogenes. Hyper activation of HDACs can lead to repression of tumour suppressor genes by removing activating acetyl marks at corresponding regulatory elements. HDAC inhibitors such as SAHA can restore the reduced expression of tumour suppressor genes in cancer. (**B**) Elevated levels of DNMTs and PRC2 can lead to expansion of suppressive methylation marks at regulatory elements that control tumour suppressor genes. Targeting DNMT and PRC activity by chemicals such as 5-Azacytidine and EZH2i, EPZ-6438, can resolve the repressed chromatin and restore the expression of tumour suppressor genes.

Chemicals that target the epigenome are among front liners of cancer therapies. Such therapies include approaches that aim at restoring the expression of tumour suppressor genes by resolving the repressed chromatin state at corresponding regulatory sites. Inhibiting DNMTs and the PRC complex (counteracting DNA and histone methylation) are among such approaches (Figure 3A). In this regard, the cytosine analogue, 5-aza-2′-deoxycytidine, has been one of the first clinically approved epi-drugs that could successfully eradicate tumour cells by inactivating the DNMTs [129]. DNMTs can be also inhibited by naturally occurring compounds such as Shikonin and non-nucleoside inhibitor RG108, both of which are known to restore the expression of tumour suppressor genes such as *PTEN* [130]. As methylated DNA can serve as a substrate for further histone methylation [68], targeting the histone methyl transferases serves as another approach for reactivating tumour suppressor genes. For example, EPZ-6438 (an EZH2 inhibitor that represses PRC2 activity) inhibits the proliferation of cancer cells in haematological malignancies [51,131,132] and induces apoptosis by restoring the expression of pro-apoptotic factors (such as *FBXO32*) [133].

In addition to histone methylation, aberrant histone acetylation is also a major influencer of suppressor genes and oncogenes expression (Figure 3B). Therefore, enhancer reprogramming via targeting histone acetylation provides a promising therapeutic strategy in cancer. One such strategy is based on the upregulation of HDACs or inhibiting the activity of HATs, leading to a loss of the acetylation at CREs associated with oncogenes [134]. Such examples include the use of small molecules, such as Comp 5, that induces SIRT1 catalytic activity, resulting in the deacetylation of H3 in glioma or using HAT inhibitors such as CCS1357 for targeting P300/CBP in prostate cancer, leading to the downregulation of *AR* and *MYC* [43,135]. The combined inhibition of epi-factors, e.g., P300/CBP (by GNE-781) and BRD4 (by OTX015), have been also applied for decommissioning oncogenic enhancers (e.g., *MYC* enhancers), and can increase the anti-tumour efficacy [136,137]. Targeting tumour-associated super enhancers also provides a promising epigenetic therapy, as addiction to oncogenic enhancers can be observed in many tumours [138].

Another strategy for targeting histone acetylation is based on using HDAC inhibitors to impede the global histone deacetylation, particularly at enhancers associated with tumour suppressor genes (Figure 3B). Such examples include the use of SAHA, an inhibitor of HDAC, that restores the expression of tumour suppressor genes which contribute to autophagy, apoptosis, and G2/M arrest in cancer cells [139]. However, the HDAC inhibitor effects are not always fully predictable due to the complex interactions between epigenetic regulators. For instance, HDAC inhibitors, such as largazole, induce a global hyperacetylation across the genome, most notably at poised enhancers. Here, a low dose of largazole increases H3K27ac and H3K9ac levels and enhances the transcriptional activity as anticipated. Surprisingly, treating cells with higher doses of HDACi strips acetylation from H3K27 residues at enhancers and super enhancers, leading to the repression of associated genes, such as MYC and AP-1, by halting the RPOL2 complex. Consequently, this unexpected global enhancer decommissioning drastically affects the proliferation of transformed cells, as they are more reliant on the super enhancer-regulated pathways [44].

Another approach for modulating oncogenic enhancers is to inhibit the commissioning TFs (or signalling effectors) alongside the general epigenetic modifiers [140]. For instance, the simultaneous targeting of BRD4 (by BETi) and oncogenic pathways, such as WNT and MAPK, in CRC inhibits the oncogenic expression of *MYC* and decreases the risk of tumour resistance [141]. For this aim, it is important to identify the dependency on key TFs by interrogating the landscape of active enhancers in tumour cells [142,143]. Profiling active enhancers was recently applied in meningioma, and it could not only stratify different tumour subtypes, but also proposed druggable enhancers and their dependencies on upstream signalling pathways [142]. The efficacy of co-inhibition strategies is more evident in tumours with hormonal dependencies. For instance, the co-inhibition of PRC2 and glucocorticoid receptor (GR) in lymphoma could drastically halt tumour proliferation by harnessing the GR addiction of oncogenic enhancers [144,145]. A similar approach applies for managing ER-α-induced enhancers in breast and endometrial tumours, where inhibiting epigenetic factors enhances the anti-tumour efficacy of hormone therapy [146,147]. However, considering the ever-changing repertoire of epigenetic regulators in cancer, there is always a risk of developing resistance. Tackling this problem requires holistic analysis to map the activity of enhancers and their associated factors through the course of tumour treatment and cancer progression.

## 4. Conclusions

In this review, we have discussed the epigenetic deregulation of transcriptional enhancers and how it fuels the cell plasticity in cancer. We discussed the spectrum of enhancer-reprogramming during EMT and lineage differentiation of adult stem cells and highlighted examples of how these cell-state transitions underlie the cell heterogeneity in tumours. As enhancers connect the upstream signalling to the downstream gene expression program, investigating the enhancer profiles in tumours can reveal the key chromatin factors that control the transcriptional programs in tumour cells. Furthermore, we covered findings of the PCAWG project on how structural variations that disrupt the sequence or the positioning of enhancers (in relation to their target genes) influence the activity of enhancers in cancer. New advances in high resolution imaging and single cell analysis (e.g by a combination of scRNA-seq, scATAC-seq, and low/ single cell ChIP-seq and DNA methylation analysis) will provide further insights into the heterogeneity of enhancer activity in different tumour subpopulations. Furthermore, the role of cancer associated polymorphisms and structural variants at enhancers remain largely unknown; new approaches using the CRISPR–Cas9 system for (epi)genetic editing will provide new methods for testing the functional contribution of non-coding variants in cancer. Finally, how tumour cells adapt their epigenenome to acquire resistant to cancer therapies, remain an important area for further investigation. Understanding this epigenetic adaptation will provide opportunities for using epigenetic drugs that target enhancers to modulate cell plasticity within the tumour (e.g., by induce differentiation in stem like tumour cells).

## Figures and Tables

**Table 1:** Epigenetic modifications and the corresponding modifiers.

Modification	Location	Modifiers		Examples of Modifier’s Contribution to Cancer
H3K27ac	Active enhancers and promoters	Writer	P300, CBP	Inactivating mutations [36,37,38], deregulated interactions [39,40], exploitation downstream of oncogenic signallings for EMT [41], expression deregulation [42,43,44]
		Eraser	HDAC1,2	
H3K4me3	Active promoters	Writer	MLL1, SETD1B	Deregulations downstream of oncogenic signalling pathways and EMT [45,46]
		Eraser	KDM2B	
H3K4me1	Poised and active enhancers	Writer	MLL3/4	Inactivating mutations [47], hijacked by oncogenic signallings [48]
		Eraser	KDM1B	
H3K27me3	Repressed and poised CpG-rich promoters and enhancers	Writer	PRC2	Inactivating mutation [49,50], deregulated expression [51]
		Eraser	KDM6	
DNA methylation	CpG-enriched promoters and genebody	Writer	DNMTs	Inactivating mutation [52], expression deregulation [53]
		Eraser	TET	
